# Efficient CRISPR/*Cas*9-mediated genome editing of phytoene desaturase in *Musa*-AAA: a critical step for genetic improvement of east African highland bananas

**DOI:** 10.3389/fpls.2025.1677409

**Published:** 2025-09-16

**Authors:** Frank Kalungi, Julius Mulindwa, Samwel Kariuki Muiruri, Valentine Otang Ntui, Abubakar Sadik Mustafa, Priver Bwesigye Namanya, Jerome Kubiriba, Leena Tripathi, Alex Barekye, Jimmy Moses Tindamanyire

**Affiliations:** 1 National Agricultural Research Laboratories, National Agricultural Research Organisation, Kampala, Uganda; 2 Department of Biochemistry and Systems Biology, College of Natural Sciences, Makerere University, Kampala, Uganda; 3 International Institute of Tropical Agriculture (IITA), Nairobi, Kenya; 4 Department of Plant Sciences, Microbiology and Biotechnology, College of Natural Sciences, Makerere University, Kampala, Uganda

**Keywords:** CRISPR/Cas9, banana, PDS, genome-editing, carotenoids, Nakitembe, guide-RNA

## Abstract

East African highland bananas (EAHBs), locally referred to as “matooke”, are an important staple crop in Uganda. The EAHBs have a triploid genome (AAA) with a large phenotypic diversity in the Great Lakes region of Africa and are challenged by both abiotic and biotic factors. The EAHBs have been improved through conventional breeding and genetic engineering though facing challenges such as genetic drag of unfavorable traits and complex regulatory processes, respectively. Therefore, a more precise approach for crop improvement such as genome editing is highly recommended. In the current study, we assessed the feasibility and applicability of the CRISPR/*Cas*9 mediated-genome editing in EAHBs. Two sgRNAs were designed from the *Nakitembe* phytoene desaturase (*PDS*) gene and used to edit the *PDS* gene in Nakitembe (NKT) and NAROBan5 (M30) cultivars. A total of 47 NKT and 130 M30 events were regenerated via *agrobacterium*-mediated transformation of banana embryogenic cell suspensions. Up to 100% and 94.6% albinism rates were observed in Nakitembe and M30 cultivars respectively with additional albino-variegated and variegated phenotypes observed in M30 only. Carotenoid analysis revealed a significant reduction of total carotenoid content in edited events with all complete albinos showing no detectable carotenoids implying that the carotenoid biosynthetic pathway was effectively disrupted. Sequence analysis revealed that all of the edited events had frameshift mutations leading to *PDS* disruption. Overall, this study presents the first report of CRISPR/*Cas*9 genome editing in EAHBs and more interestingly on a hybrid, M30 showing high precision and efficiency. This validated genome editing system provides a robust platform for targeted EAHB improvement.

## 1 Introduction

Banana (*Musa* spp.) is a perennial, herbaceous monocot cultivated both commercially and through subsistence farming across the wet tropics and sub-tropics (De Langhe et al., 2009). Edible banana varieties display diverse genomic constitutions, for instance, many sweet dessert and East African Highland bananas (EAHBs) have a triploid AAA genome, other cooking and starchy plantains and additional dessert bananas are AAB, ABB and AAAB while some seedless diploid (AA or AB) are also cultivated (Zorrilla-Fontanesi et al., 2020). The EAHBs which belong to the Lujugira-Mutika subgroup (with ‘Lujugira’ referring to a beer-type clone in Luganda) are especially vital to the food security of over 50 million people in the Great Lakes Region of Africa (GLA) (Kitavi et al., 2016).

Banana productivity is greatly constrained by biotic factors such as pests and diseases, as well as abiotic factors like water, temperature, nutrient deficiencies and light intensity. These challenges have driven efforts to develop improved cultivars. In Uganda, scientists at the National Agricultural Research Organisation (NARO) have conventionally bred and released high yielding banana hybrids - NAROBan1, NAROBan2, NAROBan3, NAROBan4 and NAROBan5 with high resistance to black Sigatoka, a fungal disease caused by *Mycosphaerella fijiensis* that leads to substantial yield loses (Tumuhimbise et al., 2018). However, the triploid nature of EAHBs hampers the production of viable gametes which creates a significant barrier to introduce new germplasm through sexual recombination-based breeding methods (Kitavi et al., 2016).

Advances in biotechnology such as marker-assisted breeding, genetic engineering, genome editing, synthetic biology, bioinformatics and systems biology offer promising strategies for developing banana varieties with enhanced traits of interest such as increased resistance to pests and diseases. For example, transgenic banana expressing rice *Xa*21 pattern recognition receptor has shown resistance to *Xanthomonas vasicola pv. musacearum* (Tripathi et al., 2014). However, the application of genetic engineering remains constrained by complex regulatory processes as well as persistent negative consumer perceptions. Meanwhile, the ever increasing availability of large biological data sets, sophisticated analytical tools and deeper understanding of biological systems has paved the way for innovative breeding approaches including genome editing.

Genome editing employs site-specific endonucleases to introduce double-stranded breaks (DSBs) at precise target sites within the DNA, which are subsequently repaired through non-homologous end joining (NHEJ) or homology directed repair (HDR) mechanisms (Borrelli et al., 2018). Key genome editing technologies include clustered regularly interspaced short palindromic repeats (CRISPR)/CRISPR-associated protein 9 (Cas9) (CRISPR/Cas9), zinc finger nucleases (ZFNs) and transcription activator-like effector nucleases (TALENs) (Mao et al., 2019). Among these, CRISPR/Cas9 genome editing has emerged as highly precise, efficient and versatile tool for genome editing across a wide range of dicotyledonous and monocotyledonous plant species (Bao et al., 2019; Hassan et al., 2021). Of the three CRISPR/Cas9 systems - type I, II, and III, the type II system derived from *Streptococcus pyogenes* is the most widely utilised. The type II system consists of two key components: the Cas9 endonuclease and a single guide RNA (sgRNA), which features a 20 nucleotide spacer sequence that directs the Cas9 protein to the target gene of interest, along with the a conserved Cas9 binding domain (Mali et al., 2013). With precise gene editing, this system introduces cuts in the DNA, allowing for the replacement, deletion, or insertion of specific sequences (Molinari et al., 2021).

Phytoene desaturase (PDS) catalyzes the desaturation of phytoene to *ζ*-carotene which is then converted into lycopene and it interacts with multiple metabolites such as abscisic acid and strigolactones (Moise et al., 2014). The *PDS* gene has been used as a marker to successfully establish and confirm genome editing in a variety of plant species like *Arabidopsis* (Qin et al., 2007), apple (Nishitani et al., 2016), cassava (Odipio et al., 2017), melon (Hooghvorst et al., 2019a), strawberry (Wilson et al., 2019), rice (Banakar et al., 2020), papaya (Brewer and Chambers, 2022), celery (Liu et al., 2022), chilli pepper (Bulle et al., 2024) and pigeon pea (Senthil et al., 2025).

Despite their significance as a staple food in Uganda and across the Great Lakes region of Africa, there are currently no published reports on genome editing in EAHBs. Previous studies have demonstrated successful CRISPR/Cas9-mediated editing of the *PDS* gene in other banana cultivars, resulting in high rates of variegation; however, these outcomes suggest that editing efficiency may vary between cultivars (Ntui et al., 2020). This underscores the need to assess the feasibility of genome editing specifically in EAHBs. In the current study, we identified the *PDS* gene from the genome of the EAHB cultivar ‘Nakitembe’, which we sequenced in-house, and used it as a target to evaluate the efficiency and applicability of CRISPR/*Cas*9 in EAHBs. Our results provide a foundation for the precise genetic manipulation of key agronomic traits in EAHBs, with potential implications for improving food security in the GLA.

## 2 Results

### 2.1 Target sites in the NKT *PDS* gene and sgRNA design

**Figure 1:**
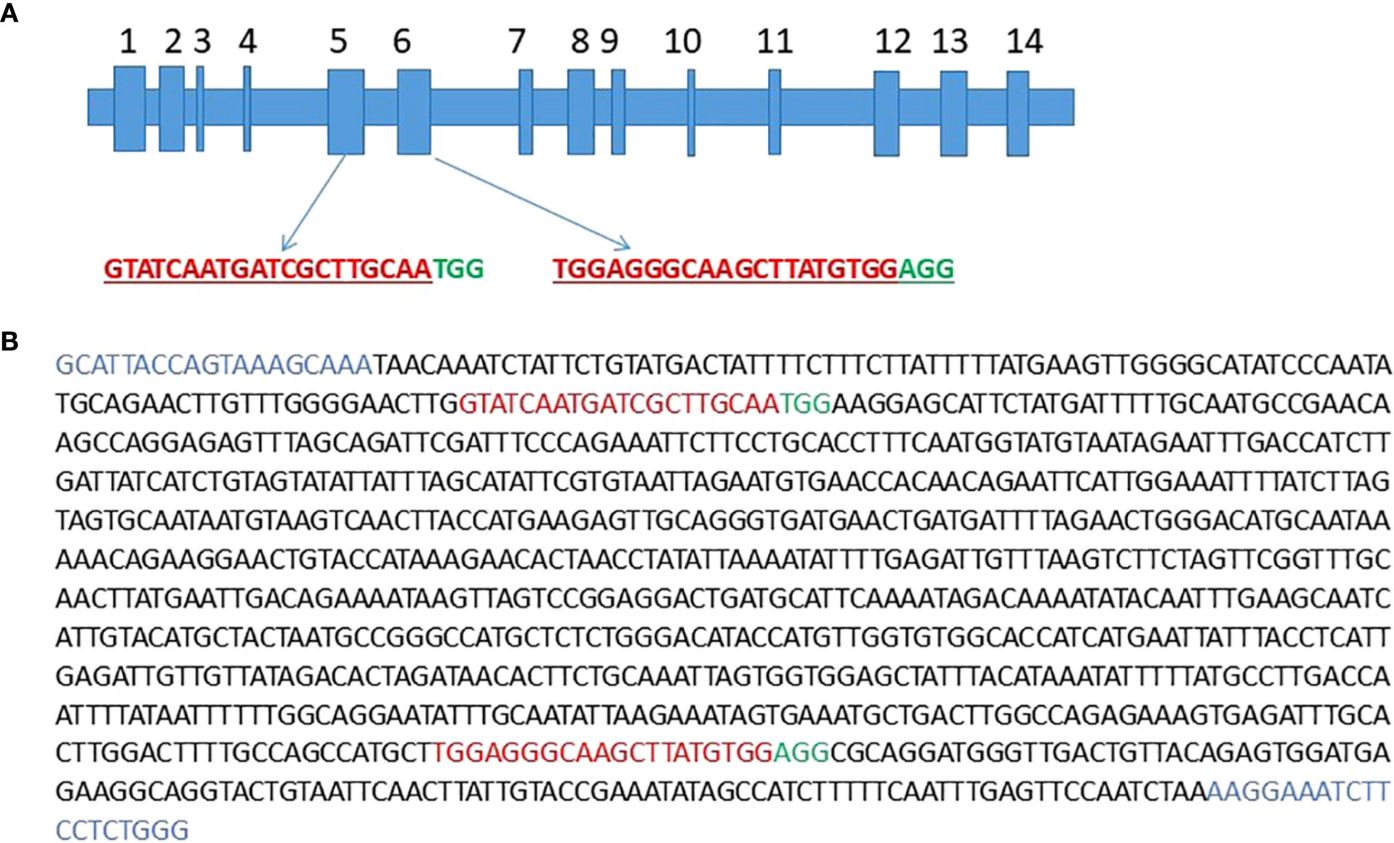
Exon 5 and 6 of Ma08_t16510.2 were used to design gRNA1 and gRNA2 respectively **(A)**. Partial fragment of NktPDS showing positions for sgRNA design **(B)**. The sgRNAs, protospacer adjacent motifs (PAMs) and band-shift PCR primers are in represented as red, green and blue respectively.

The 4,006 bp sequence referred to as Nkt*PDS* was mined from the full genome sequence of the NKT and it was BLASTed against *M. acuminata DH. Pahang* (v4) genome using the Banana Genome Hub (https://banana-genome-hub.southgreen.fr/). Comparative analysis with *M. acuminata DH Pahang* (v2) revealed the gene model Ma08_t16510.2 had 100% identity, and it was therefore selected for exon mapping (**Figure 1**). Ma08_t16510.2 had 14 exons and to maximise the likelihood of producing non-functional *PDS* transcripts, the first six exons were selected to create an intermediary sequence named “six NKT”. To rule out SNPs, an alignment between Nkt*PDS* and “six NKT” was performed and the first 121 bp conserved region of Nkt*PDS* was identified and chosen to design the two sgRNAs. These sgRNAs were synthesised as oligonucleotide pairs (OP7/OP8 and OP9/OP10) with the appropriate adaptor sequences, as detailed in **Supplementary Information Table 1**.

### 2.2 CRISPR/Cas9 vector plasmid

**Figure 2:**
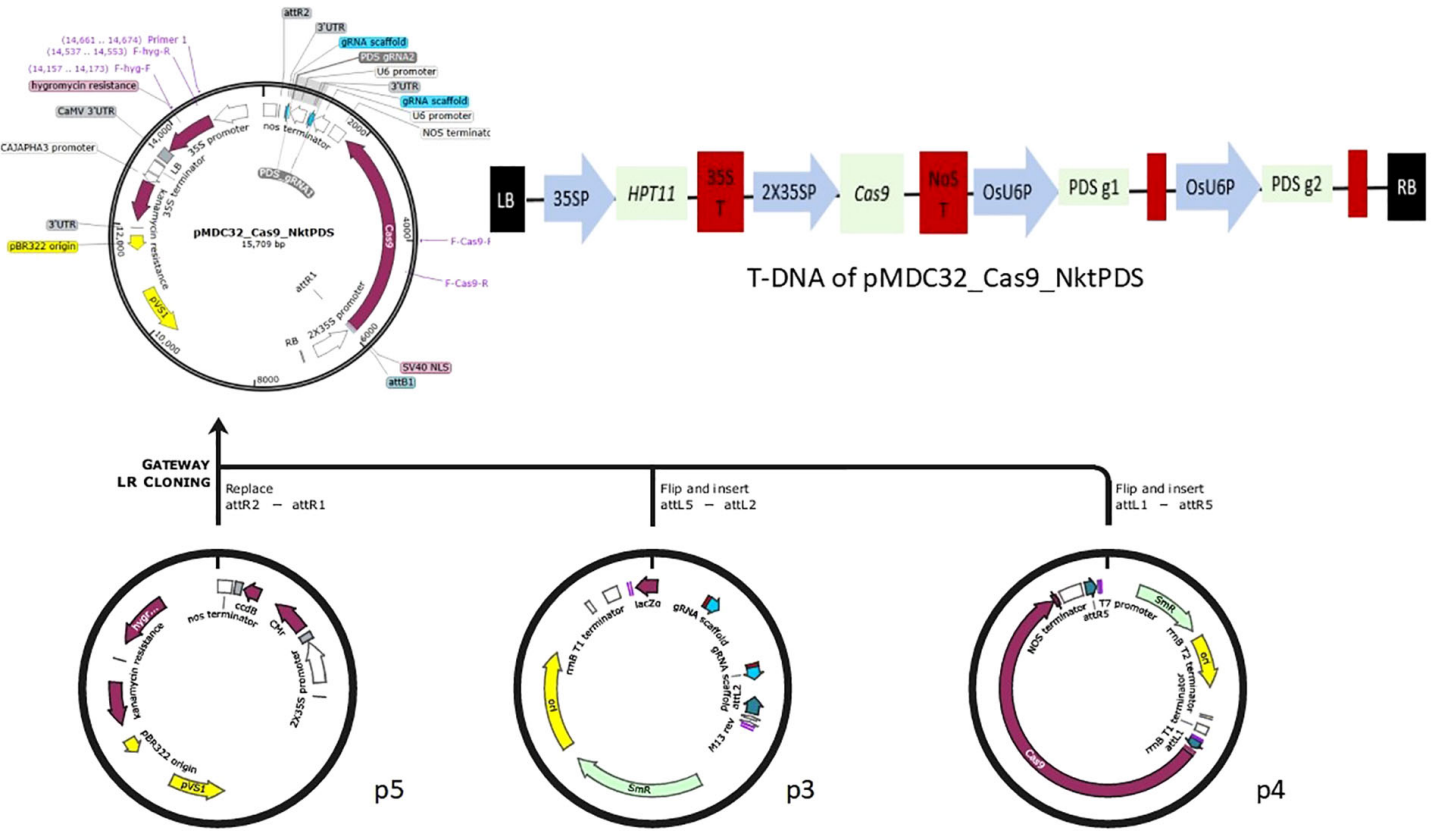
Multiplex assembly of sgRNAs and Cas9 into pMDC32 to form a binary construct, pMDC32_Cas9_NktPDS. p3; pYPQ142_PDS_g1_g2, p4; pyPQ167 and p5; pMDC32.

Two sgRNAs were designed and individually cloned into the sgRNA expression plasmids pYPQ131C and pYPQ132C. These were then multiplexed into pYPQ142 via Golden Gate cloning. The resulting cassette was recombined with a Cas9 entry vector pYPQ167 and the binary vector pMDC32 to generate the final construct, pMDC32_Cas9_NktPDS (**Figure 2**). The pMC32_Cas9_NktPDS was first transformed into *E. coli* DH5α for propagation and subsequently into *A. tumefaciens* strain *AGL1* for banana transformation.

### 2.3 Regeneration of EAHB plants with gene-edited *PDS* gene

**Figure 3:**
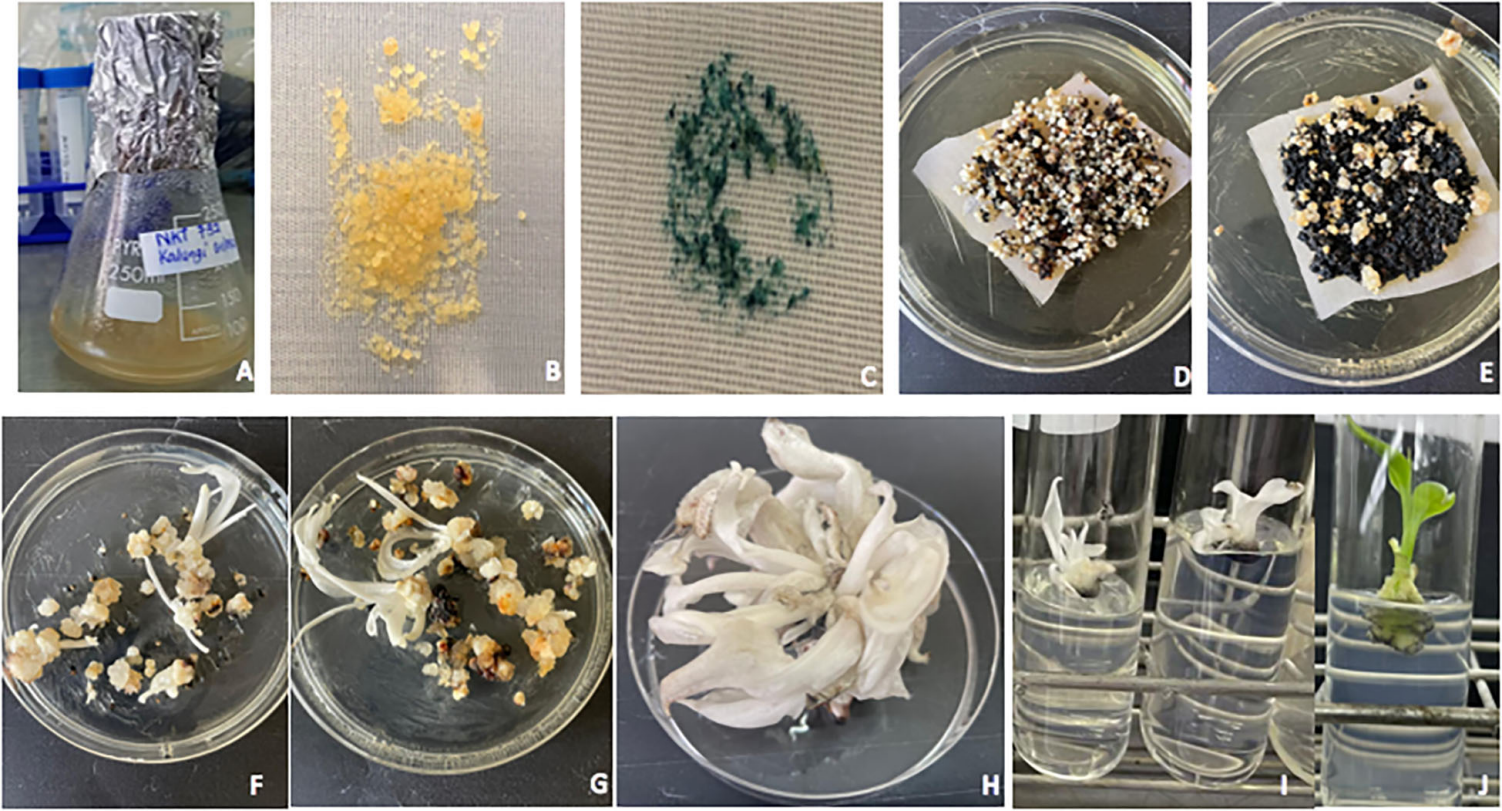
Genetic transformation and regeneration of *in vitro* gene-edited banana plantlets. Banana ECS in MA2 media prior to transformation **(A)**, Co-cultivation of ECS on MA3 media **(B)**, Histochemical GUS assay of cells transformed pUBI: GUS **(C)**, selection of transformed cells on MA3 media **(D)**, embryo development on MA3 media **(E)**, embryo germination and shoot development on MA3 media **(F, G)**, individual gene-edited plants with “albino” phenotype **(H, I)** and wild-type control **(J)** on proliferation media.

Banana ECS lines NKT-732 and M30-885 were *Agrobacterium*-transformed with pMDC32_Cas9_NktPDS and pUBI: GUS, and sub-cultured on selective media for plant regeneration (**Figure 3**). Histochemical GUS assays on cells transformed with pUBI: GUS construct showed blue-staining, confirming successful transformation of banana ECS. A total of 47 and 130 gene-edited events were regenerated for NKT and M30 respectively. Notably, the edited events exhibited slower growth compared to wild-type. More interestingly, M30 edited events began browning and wilting after two weeks on proliferation media, whereas browning in NKT was observed after one month, suggesting cultivar-specific physiological responses despite sharing the same genome group. As a result of browning, gene-edited events were frequently sub-cultured on proliferation media every month to maintain viability. A large number of these events were also kept in the dark to minimise photo-oxidation, thereby reducing oxidative damage and prolonging their survival in culture.

### 2.4 Molecular characterisation of gene-edited events

#### 2.4.1 PCR analysis to confirm integration of *Cas*9 and *hpt*II genes

**Figure 4:**
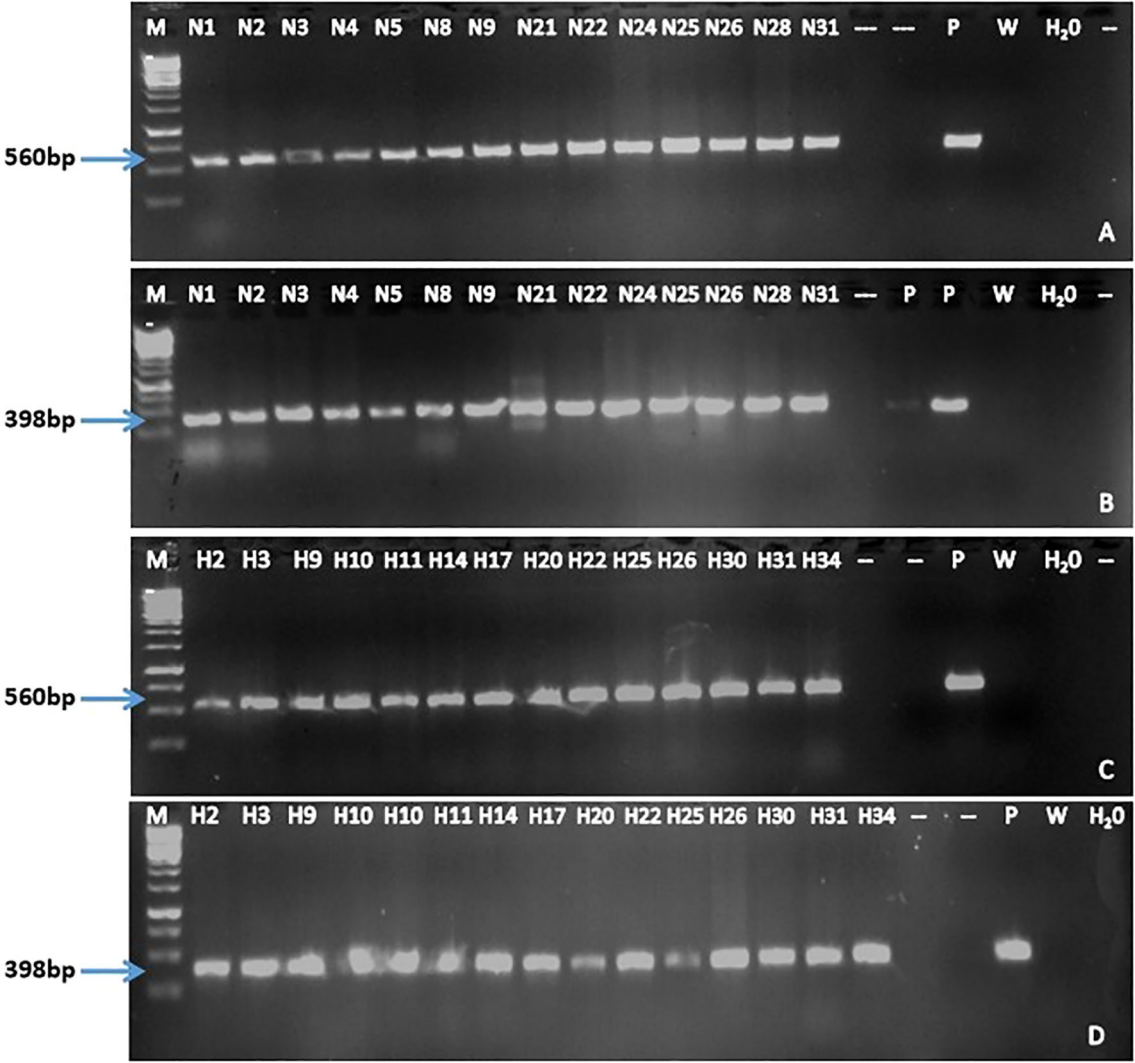
Detection of the Cas9 and hptII genes in selected PDS gene-edited EAHB banana lines by PCR. Presence of Cas9 **(A)** and hptII **(B)** in NKT lines while Cas9 **(C)** and hptII **(D)** genes in M30 lines. M; GeneRuler 1 kb DNA Ladder (Thermo Scientific™), W; wild type, P; pMDC32_Cas9_NktPDS, and H2O; water control.

End-point PCR was performed to confirm the integration of *Cas*9 and *hpt*II genes using primer pairs OP1/OP2 and OP3/OP4 respectively. All selected gene-edited lines from both NKT and M30 were confirmed positive for *Cas*9 and *hpt*II genes, with amplicons of 560 and 398 bp respectively (**Figure 4**). As expected, none of the wild-types tested positive for either gene.

#### 2.4.2 PCR analysis to detect band shift

**Figure 5:**
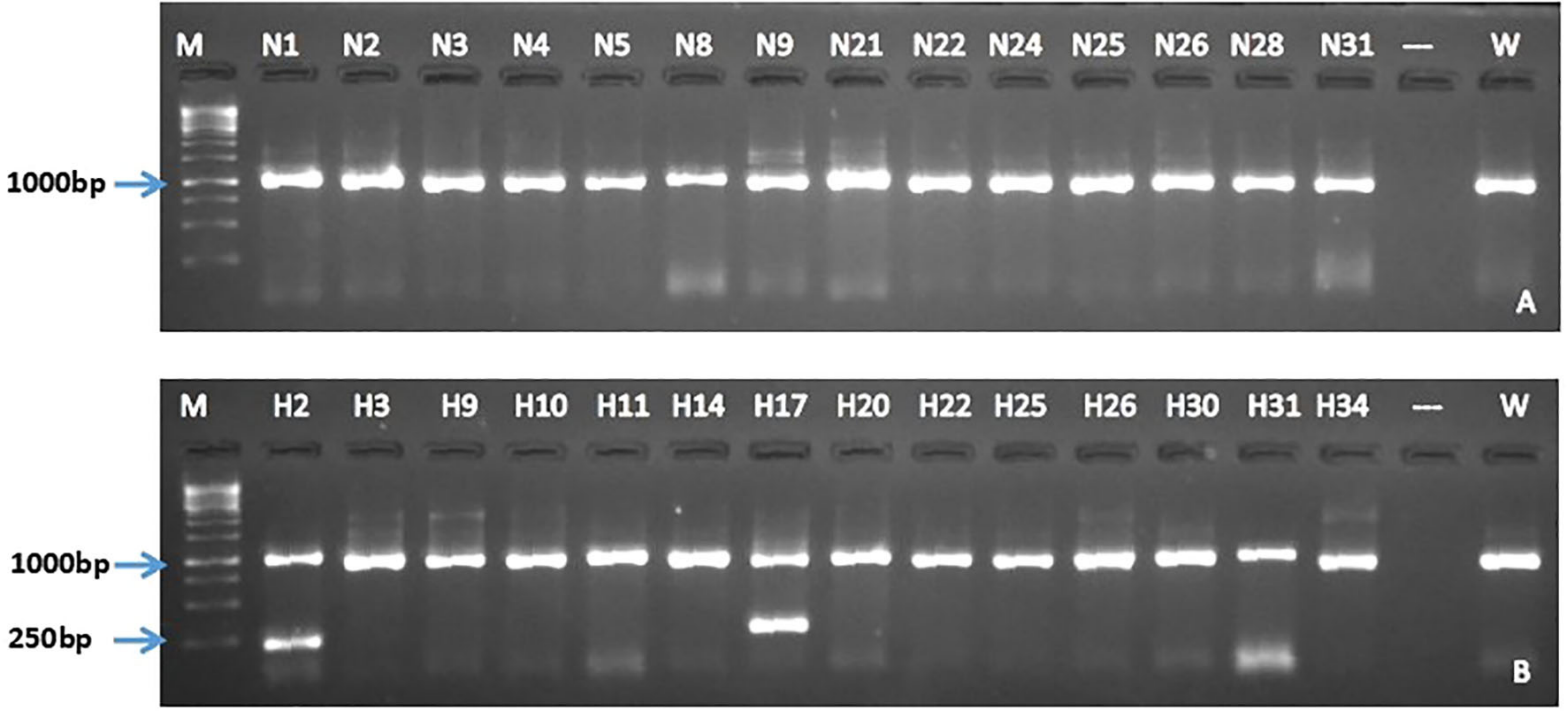
Detection of band shift in the regenerated events of NKT **(A)** and M30 **(B)** by PCR. M; GeneRuler 1 kb DNA Ladder (Thermo ScientificTM), W; wild-type.

To enhance the precision of *PDS* gene editing, two sgRNAs spaced 719 bp apart were introduced into NKT and M30 cultivars. Gene-specific primers OP5 and OP6 were used in band-shift PCR to detect size differences between wild-type and gene-edited events. All regenerated events previously confirmed for *Cas*9 and *hpt*II integration (**Figure 4**) were selected and subjected to this analysis. In wild-type plants, primers OP5/OP6 amplify a 956 bp fragment. However, a successful dual-sgRNA-mediated deletion would result in a shortened amplicon of approximately 237 bp due to a 719 bp deletion. All the edited NKT events produced a 956 bp identical to the wild-type, suggesting only small indels (**Figure 5A**). Similarly, most M30 edited events showed a 956 bp band, except for events H2 and H17, which displayed an additional but smaller amplicon. Event H2 exhibited the expected band shift indicating that both sgRNAs were successfully cleaved simultaneously (**Figure 5B**).

#### 2.4.3 Detection of mutations by Sanger sequencing

Four gene edited M30 events (H2, H3, H17, H30), two gene-edited NKT events (N2, N25) and a wild-type were selected based on band shift PCR patterns and phenotype for mutational analysis by sequencing (**Table 1**). M30 events showed either deletions or insertions at sgRNA target sites. H2 showed a biallelic large deletion of 724 bp suggesting dual homozygous editing by both sgRNAs. H3 and H17 showed small deletions of 1 bp and 46 bp at gRNA2 (g2) and gRNA1 (g1) sites respectively suggesting homozygous edits while H30 showed both edited allele (+1 bp at g1) suggesting a heterozygous mutation. NKT lines showed various mutations with N2 displaying 98 bp deletion at g2 at one allele and small indels (+1/-2 bp; g1/g2) in another allele indicating a heterozygous mutation. N25 had a small deletion of 2 bp at g2 indicating a homozygous state. Both NKT and M30 events showed frameshift mutations, with only N2 and H2 showing mutations at both of the target sites.

**Table 1:** Sanger sequencing of selected NKT and M30 gene-edited lines.

WT	GTATCAATGATCGCTTGCAATGG..719..TGGAGGGCAAGCTTATGTGGAGG	WT	sgRNA
H2	GTATCA-----------------..719..TGG--------------------	-724/-60	g1/g2
H3	GTATCAATGATCGCTTGCAATGG..719..TGGAGGGCAAGCTTAT-TGGAGG	-1	g2
H17	-----------------CAATGG..719..TGGAGGGCAAGCTTATGTGGAGG	-46	g1
H30	GTATCAATGATCGCTTGCAATGG..719..TGGAGGGCAAGCTTATGTGGAGG GTATCAATGATCGCTTCGCAATGG.719..TGGAGGGCAAGCTTATGTGGAGG	+1	g1
N2	GTATCAATGATCGCTTGCAATGG..719..TGGAGG----------------- GTATCAATGATCGCTTGACAATGG.719..TGGAGGGCAAGCTTA--TGGAGG	-98 +1/-2	g2 g1/g2
N25	GTATCAATGATCGCTTGCAATGG..719..TGGAGGGCAAGCTTA--TGGAGG	-2	g2

WT; wild-type, red nucleotides; target sites, “-”; deletion, light blue nucleotides; insertions and purple nucleotides; PAM.

### 2.5 Phenotypic characterization of gene-edited East African highland banana plants

**Figure 6:**
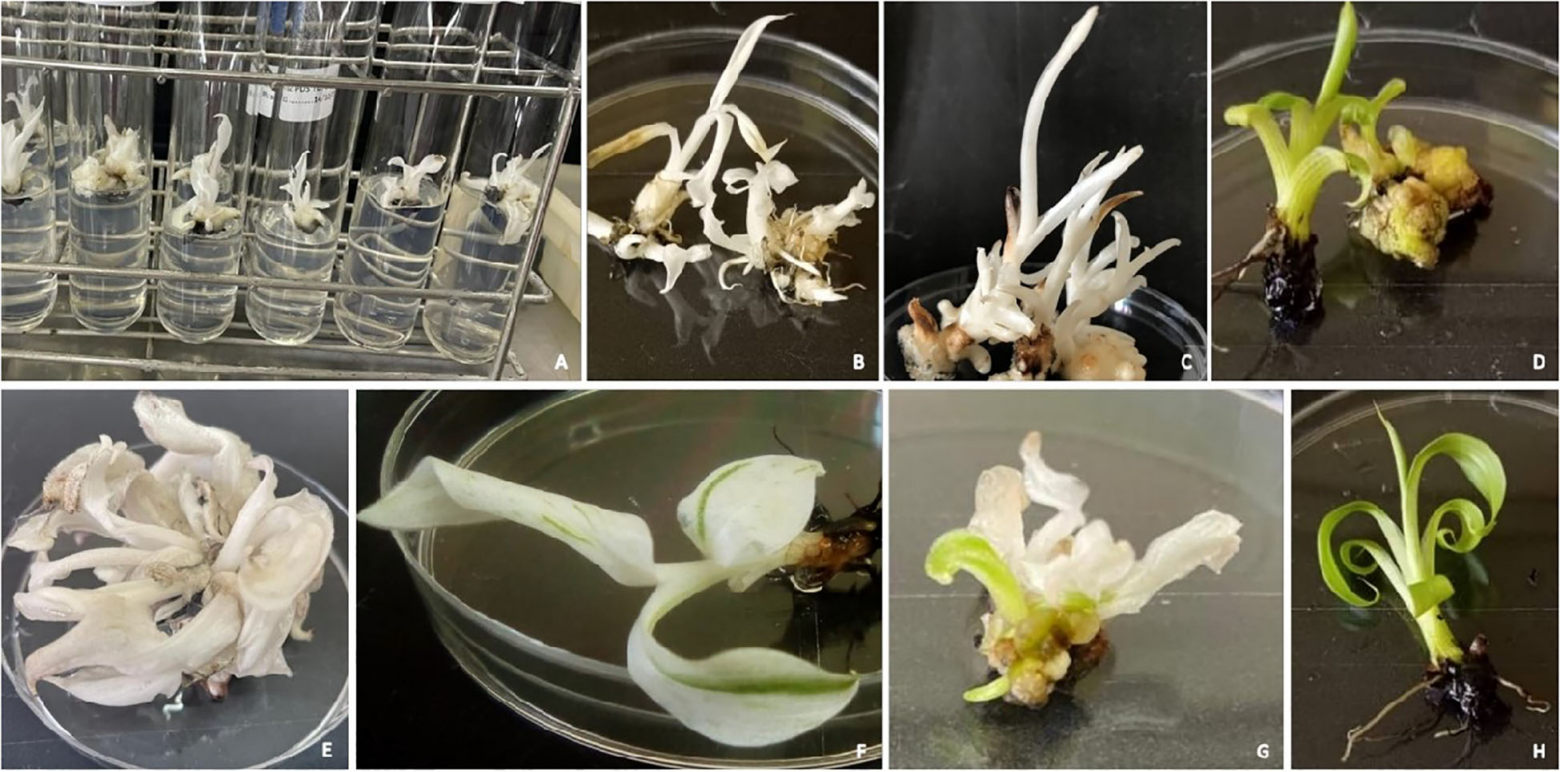
Phenotypic characteristics of regenerated events. NKT; albino **(A–C)** and wild-type control **(D)**, M30; albino **(E)**, albino-variegated **(F)**, variegated plant **(G)** and wild-type control **(H)**.

The regenerated gene-edited events exhibited various phenotypes, including completely white (albino) shoots, white shoots with green stripes (albino-variegated) and mixed green and white shoots (variegated), as illustrated in **Figure 6**. In contrast, wild-type plants of both M30 and NKT exhibited uniform green shoots. Among the M30 gene-edited lines, 123 lines were albino, 6 were albino-variegated and 1 was variegated. All 47 gene-edited NKT lines were completely albino (**Table 2**). Notably, no non-transformed escape events were observed in either cultivar. The gene-edited events also exhibited dwarfism and, in some instances, produced very small shoots upon subsequent sub-culturing. A subset of both gene-edited and wild-type plants was selected for further analysis of carotenoid profiles and amounts.

**Table 2:** Phenotypic characteristics of regenerated gene-edited events.

Cultivar	#Events	Phenotypic trait (%)			Mutation (%)
		Albino	Albino- variegated	Variegated	
NKT	47	100	–	–	100
M30	130	94.6	4.6	0.8	100

### 2.6 Carotenoid analysis and quantification

**Figure 7:**
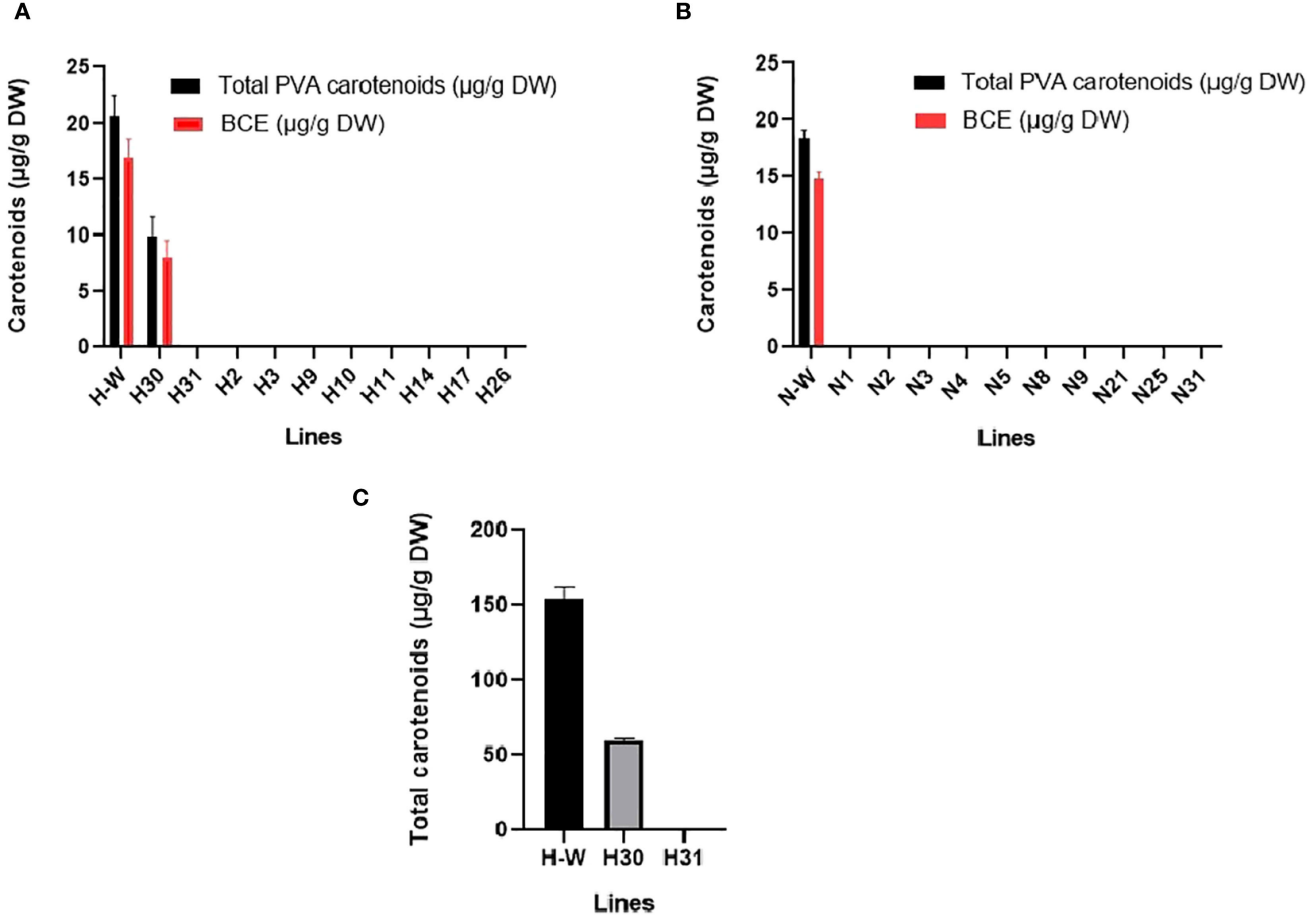
Total PVA carotenoids and BCE content of selected M30 **(A)** and NKT **(B)** lines. Total carotenoids of selected M30 lines **(C)**. H-W; wild-type control, H30; variegated, H31; albino-variegated. All data are presented as presented as mean± SD. Statistical analysis was performed using one way ANOVA revealing significant differences among groups (P=0.0001). Dunnett’s multiple comparisons test indicated that both H30 and H31 had significantly lower carotenoid levels compared to the H-W at a significance level of P < 0.05 with adjusted P-values of 0.0004 and 0.0001 respectively.

Ten gene-edited lines and one wild-type line per cultivar were selected for HPLC analysis based on band-shift PCR results and observed phenotypes. For NKT, the selected events included N-W, N1, N2, N3, N4, N5, N8, N9, N21, N25 and N31 while M30 were H-W, H2, H3, H9, H10, H11, H14, H17, H26, H30 and H31. HPLC analysis at 450 nm revealed that all gene-edited lines from both NKT and M30 lacked detectable levels of carotenoids, except for one variegated M30 event (H30) and one albino-variegated event (H31). In banana leaf tissue, three major carotenoid peaks - lutein, *α*-carotene and *β*-carotene were detected with retention times between 20 to 25 minutes. As expected, the M30 wild-type event (H-W) showed higher carotenoids than the variegated event (H30). Notably, in H31, lutein was the only carotenoid detected indicating a complete disruption of *α*- and *β*-carotene biosynthesis and significant reduction in lutein production. Moreover, H-W had a higher total carotenoid content than the NKT wild-type (N-W), suggesting physiological differences between the two cultivars. The total carotenoid content, PVA carotenoids and BCE of selected NKT and M30 lines are summarised in **Figure 7** and **Supplementary Table 2**.

## 3 Discussion

New plant breeding technologies like transgenesis, RNAi silencing, and genome editing offer transformative potential for crop improvement, including in banana. Genome editing, particularly using CRISPR/Cas9, introduces precise mutations similar to those occurring naturally or through traditional mutagenesis. It is increasingly favoured for its efficiency and potential to produce superior crop traits. The *PDS* gene has commonly been used as a visual marker to establish CRISPR/Cas9 systems in various crops such as *Arabidopsis* (Qin et al., 2007), cassava (Odipio et al., 2017), melon (Hooghvorst et al., 2019a), strawberry (Wilson et al., 2019) and rice (Banakar et al., 2020). In the current study, the *PDS* gene was similarly used to validate and evaluate the feasibility and efficiency of genome editing in East African Highland Bananas (EAHBs).

In the current study, two sgRNAs targeting the *PDS* gene of the NKT cultivar, spaced 719 bp apart, were multiplexed with the *Cas9* gene and delivered into embryogenic cell suspensions (ECS) of both NKT and M30 cultivars via *Agrobacterium*-mediated transformation. A total of 100% and 94.6% of regenerated plants exhibited a complete albino phenotype in NKT and M30 cultivars, respectively. This contrasts with earlier findings by Ntui et al. (2020) and Naim et al. (2018) who reported albino phenotypes in 67 - 94% of Sukali Ndiizi and Gonja Manjaya events and in 63% of Cavendish events. Notably, albino-variegated and fully variegated phenotypes were observed only in M30, likely because the sgRNAs were specifically designed based on the NKT *PDS* gene sequence, underscoring the importance of cultivar-specific genomic information in sgRNA design. Kaur et al. (2018) similarly reported that variegation could result from monoallelic or biallelic edits, underlying genome heterozygosity, or variation in sgRNA/Cas9 activity. Furthermore, *Agrobacterium*-mediated delivery of plasmid-based CRISPR/Cas9 systems may lead to ongoing gene-editing, potentially producing chimeric plants composed of cells with different mutational profiles (Zhang et al., 2019).

Gene-edited plants exhibited slower growth and earlier browning, particularly in M30, consistent with phenotypes observed in other species following *PDS* disruption. Loss of carotenoid biosynthesis due to *PDS* knockout likely caused reduced carotenoids such as lutein, *α* and *β* carotene, and abscisic acid levels and increased chloroplast dysfunction and oxidative stress which ultimately impairs plant growth. The gene-edited plants survived only briefly on proliferation media and required frequent sub-culturing, suggesting possible homozygous mutations, as reported in similar studies in celery (Liu et al., 2022), *Rehmannia glutinosa* (Li et al., 2021), highbush blue berry (Vaia et al., 2022), melon (Hooghvorst et al., 2019b) and banana (Kaur et al., 2018).

Carotenoid profiling of the gene-edited events revealed no detectable carotenoids in completely albino plants from both M30 and NKT cultivars. The albino-variegated M30 event (H31) contained only lutein, while the variegated event (H30) exhibited significantly reduced carotenoid levels compared to the M30 wild-type (H-W) (**Figure 4**). These findings are consistent with previous studies such as Kaur et al. (2018), which showed that disruption of the *PDS* gene in *Rasthali* leads to pigment loss. Furthermore, the wild-type NKT plants exhibited lower total carotenoids, β-carotene equivalents (BCE), and pro-vitamin A carotenoids (PVA) compared to M30 wild-type plants (**Figure 7**). This aligns with findings by Amah et al. (2019), who reported that total carotenoid content among *Musa* genotypes ranged from 1.45 µg/g in hybrid 25447-S7 R2P8 to 36.21 µg/g in *Musa acuminata* cultivar ITC.0601 Hung Tu, with an average of 8.00 µg/g fresh weight. The observed differences between NKT and M30 may reflect underlying genetic diversity influencing the expression and regulation of genes involved in carotenoid biosynthesis and storage.

In this current study, PCR analysis using gene-specific primers confirmed stable integration of *Cas9* and *hptII* genes in all gene-edited lines, while wild-types showed no amplification as expected (**Figure 4**). Band-shift PCR analysis revealed that all edited events in both cultivars produced an amplicon of 956 bp similar to wild-type with exception for event H2 and H17 (**Figure 5**). Sanger sequencing of selected gene-edited lines revealed frameshift mutations and a 100% mutation efficiency in both cultivars. The frameshift mutations were responsible for the disruption of the *PDS* gene of EAHBs leading to albino and variegated phenotypes. The gene-edited lines generated in the current study displayed either insertions or deletions with the latter having the highest frequency. Sequencing revealed that about 66.67% of the gene-edited lines (H2, H3, H17, N25) in both cultivars had homozygous mutations while others (H30, N2) had heterozygous mutations. In contrast, Ntui et al. (2020) reported that about 50% of both Sukali Ndiizi and Gonja Manjaya gene-edited events had homozygous mutations. Our results indicate successful activation of the non-homologous end joining (NHEJ) repair pathway with the large deletion (-724 bp) in H2 confirming effective dual cleavage by the sgRNAs. The NHEJ is a preferred mechanism in plant genome editing, as it enables targeted modifications without introducing foreign DNA, an important consideration for GMO acceptance (Ishii and Araki, 2017). Notably, a similar approach by Naim et al. (2018) using two sgRNAs to target *PDS* in cavendish banana which belongs to the genome group (AAA), failed to produce the expected band shift in any edited events. This might be due to the fact that Naim et al. (2018) used a human-codon optimised *Cas*9 under the expression of either a maize polyubiquitin 1 or a single CaMV 35S promoter which likely leads to moderate Cas9 abundance as compared to the double CaMV 35S promoter used in this current study. In addition, the authors used a rice U3 promoter for sgRNA expression which may be less effective in monocots as compared to OsU6 promoter. The OsU6 promoter was found to induce high sgRNA expression in rice than the OsU3 promoter (Mikami et al., 2015). Additionally, majority if not all related studies have used *in silico* sequences of the *PDS* gene but the current study used one derived from a full genome sequence of the “Nakitembe” cultivar.

In the current study, both sgRNAs were effective in both cultivars with g1 being the most effective. However, only two gene-edited lines (H2 and N2) showed mutations at both sgRNA target sites. The differences in sgRNA efficiency at target sites could be attributed several factors including sgRNA structural elements such as the GC content, nucleotide composition and stem loop formation (Bruegmann et al., 2019; Liang et al., 2016). Cas9 sliding on PAM sites has been reported to impact sgRNA activity and efficiency (Corsi et al., 2022). The search time taken by the sgRNA/Cas9 complex to locate the target site has been reported to influence sgRNA activity across different species (Moreb and Lynch, 2021). Additionally, interaction of the sgRNA/Cas9 complex at the target site influences DSB repair pathways with ultimately determines editing outcomes (Liu et al., 2022). The variation in sgRNA effectiveness across cultivars has been reported in various plants such as hot pepper (Park and Kim, 2023) and water melon (Zhao et al., 2025). The differences in editing efficiency of sgRNAs across cultivars can be due to genome differences among cultivars (Ntui et al., 2020).

Noteworthy, sequencing of H17 in this present study consistently produced a 46 bp deletion yet the event displayed two bands from band shift PCR. This implies that the sequencing primer predominantly amplified the wild-type like allele leaving out the other allele with a larger deletion, which is a common limitation of direct PCR sequencing. Therefore, more efficient sequencing approaches such as sequencing of cloned products or next generation sequencing are recommended in scenarios where direct PCR sequencing may not resolve mixed alleles in the same event. Overall, the dual-sgRNA strategy used in the current study effectively mediated genomic modifications which may be important for disruption of regulatory or coding regions. The absence of wild-type alleles in most lines highlights the high editing efficiency of the CRISPR system used in the current study.

The gene-edited products developed in this present study used *Agrobacterium-*mediated banana transformation protocol and thus are still considered as traditional genetically modified plants in many regulatory regimes. This is because CRISPR/Cas construct components such as selectable markers and Cas genes are often integrated into the plant genome which complicates biosafety regulations (Lokya et al., 2025). Notably, initiatives in our laboratory at NARO - Uganda are underway to develop transgene-free approaches based on a protoplast-based transformation system where the *Cas9*-sgRNA ribonucleoprotein (RNP) complex can be delivered using polyethylene-glycol. Since the Cas9 protein and gRNAs are not integrated into the plant genome they are eventually degraded or lost after editing leaving no foreign components in the plant’s genetic material. This eventually leads to production of transgene-free bananas. Interestingly, polyethylene glycol-mediated protoplast transfection systems for CRISPR/Cas plasmids and RNP complexes have been successfully established in banana (Lakhani et al., 2025; Leh et al., 2023; Wu et al., 2020). However, regeneration of gene edited plants from banana protoplasts still remains a major challenge. Nevertheless, transgene-free plants have already been produced in other species, for example, canker-resistant *Citrus sinensis* (Su et al., 2023) and *Solanum peruvianum* (Lin et al., 2022). The successful establishment of a banana protoplast to plant regeneration system combined with the CRISPR/Cas9 system will accelerate development of improved transgene-free EAHBs and even dessert bananas with wide range of target traits.

## 4 Conclusions

This study presents the first successful report of genome editing in East African Highland Bananas (EAHBs) including the hybrid cultivar NAROBan5 (M30), marking a significant milestone in banana genetic improvement. As a proof of concept, the validated CRISPR/Cas9 system demonstrated its ability to induce large genomic deletions highlighting its potential for targeted gene modifications in banana. This system provides a robust platform for genome-wide screening, functional gene characterisation and trait discovery, all of which are critical for developing improved EAHB varieties. Importantly, EAHBs are a major staple and income source for smallholder farmers in East and Central Africa. The ability to precisely edit genes opens avenues for developing East African highland banana cultivars with enhanced resistance to pests and diseases, improved yield, and superior nutritional value traits that directly address the needs of low-income farming communities.

## 5 Materials and methods

### 5.1 Plant material

Embryogenic cell suspensions of EAHB cv. ‘Nakitembe’ (NKT) and hybrid NAROBan5 (M30) developed by and maintained at the National Agricultural Research Laboratories were used in the current study.

### 5.2 Target gene and sgRNA design

A 4.01 kb Illumina consensus sequence of the *PDS* gene was obtained from the full genome sequence of EAHB cv. ‘Nakitembe’ (NKT). This sequence was then BLASTed against the *Musa acuminata DH. Pahang* (v4) genome using the Banana Genome Hub (https://banana-genome-hub.southgreen.fr/). The gene model Ma08_t16510.2, which had 100% sequence identity, was selected as the best-matching hit in the *M. acuminata DH. Pahang* reference genome. Using the Ma08_t16510.2 gene model, the first six candidate exons were identified, merged and aligned with NKT *PDS* consensus sequence using multiple sequence alignment (MultAlin) Software (http://multalin.toulouse.inra.fr/multalin/). A 121 bp sequence without single-nucleotide polymorphisms (SNPs) between the NKT and Pahang sequences was identified and selected for guide RNA (gRNA) design. Two single guide RNAs (sgRNAs) were generated using Integrated DNA Technologies’ Custom Alt-R^™^ CRISPR-Cas9 guide RNA design tool (www.idtdna.com): the first sgRNA (gRNA1: 5’ -GTATCAATGATCGCTTGCAA-3’) and second sgRNA (gRNA2: 5’-TGGAGGGCAAGCTTATGTGG-3’) targeting the fifth and sixth exons respectively. A guanine (G) was added to the 5’ end of forward strand and cytosine (C) to the 3’ end of the reverse complement of each sgRNA as a requirement for U6 promoter-driven expression. Additionally, adaptor sequences (‘GTGT’ forward strand and ‘AAAC’ for the reverse complement) were appended to facilitate cloning into vectors using the *BsmBI* restriction enzyme. The final oligonucleotides, including gRNA1_PDS and gRNA2_PDS along with their reverse complements (**Supplementary Table 1**), were synthesised by Macrogen Europe (https://order.macrogen-europe.com/).

### 5.3 Construction of CRISPR/Cas9 vector

The CRISPR/Cas9 construct, pMDC32_Cas9_NktPDS was developed according to Lowder et al. (2015). Briefly, the gRNA expression vectors pYPQ131C and pYPQ132C were linearised using *BsmB*I to produce 4 bp overhangs. The linearised products were purified using QIAquick^®^ PCR Purification Kit (Qiagen) according to manufacturer’s instructions. The forward and reverse oligos of the respective sgRNAs were phosphorylated and annealed using T_4_ polynucleotide kinase. The phosphorylated and annealed sgRNA oligo products, gRNA1 and gRNA2, were then ligated into linearised pYPQ131C and pyPQ132C using T_4_ DNA ligase to form pYPQ131C_gRNA1 and pYPQ132C_gRNA2 respectively. The ligated products were later transformed into *E. coli* strain DH5*α*, selected on LB medium with 50 µg/mL tetracycline. Colonies were selected and cultured in liquid LB medium from which plasmid DNA (pDNA) was extracted and verified for integrity by Sanger sequencing. Colonies with the right insert were selected after which the two gRNAs were assembled into the Golden Gate recipient and Gateway entry vector, pYPQ142 by digestion with *Bsa*I and ligation using T_4_ DNA ligase. The Golden Gate products were also transformed into chemically competent *E. coli* DH5α with spectinomycin (100 µg/mL). The pDNA of selected colonies was extracted and verified by colony PCR and restriction digestion using *Eco*RI-HF^®^ and *Nco*I-HF. A positive plasmid from the Golden Gate assembly above and the *Cas*9 entry vector (pYPQ167) were then cloned together into the Gateway binary vector (pMDC32) (Curtis and Grossniklaus, 2003) in a recombination reaction using Invitrogen™ Gateway™ LR Clonase™ II Enzyme mix (Invitrogen). The Gateway multiplex assembly reaction was then transformed in *E. coli* DH5α and cells were selected on LB agar and broth containing kanamycin (50 µg/mL). Transformed clones were verified by isolating pDNA and restriction digestion using *Kpn*I. The plasmid of the final CRISPR/Cas9 binary vector pMDC32_Cas9_NktPDS has *hpt* gene as an *in planta* selection marker, *Cas*9 gene and the two gRNAs, each driven by the rice Pol III promoter, OsU6. The *Cas*9 gene used in this plasmid is plant codon optimised and is regulated by double CaMV35S promoter.

The pMDC32_Cas9_NktPDS pDNA was transformed into *Agrobacterium tumefaciens* strain AGL1 as described by Wise et al. (2006) and transformed colonies selected on LB agar supplemented with carbenicillin (100 µg/mL), rifampicin (25 µg/mL) and kanamycin (50 µg/mL) were confirmed using colony PCR. Cultures of the validated colony of *A. tumefaciens* strain AGL1 with pMDC32_Cas9_NktPDS were prepared for transformation of banana ECS while glycerol stocks were prepared and maintained at ^­^80°C for long-term storage.

### 5.4 *Agrobacterium*-mediated transformation and regeneration of banana

The ECS lines of NKT (NKT-732) and M30 (M30-885) were transformed with vector pMDC32_Cas9_NktPDS using a modified *Agrobacterium*-mediated protocol by Khanna et al. (2004). Briefly, a single colony of *A. tumefaciens* containing pMDC32_Cas9_NktPDS binary vector plasmid was grown for 3 days in LB broth containing Carbenicillin (100 µg/mL), Rifampicin (25 µg/mL) and Kanamycin (50 µg/mL). Another colony of AGLI containing the pUBI: GUS construct harbouring *uidA* gene encoding β-glucuronidase enzyme (GUS) under the control of maize ubiquitin promoter was grown under similar conditions. For each construct, a 5 mL *Agrobacterium* starter-culture was transferred into fresh 20 mL of LB broth and grown for 24 hr under the same conditions. Each of the Agro-cultures was transferred into sterile Falcon^®^ 50 mL high-clarity polypropylene (PP) conical centrifuge tubes (Corning) and centrifuged at 6,000 rpm for 10 min at room temperature. The resultant pellet was resuspended in 25 mL TMA1 media supplemented with acetosyringone (200 μM). Shaking was done for 2.5 hr at 70 rpm and 25°C and its OD_600_ determined using Jenway™ Genova Plus Life Science spectrophotometer. A settled cell volume of 4 mL banana cells were transferred to independent Falcon^®^ 50 mL high-clarity polypropylene (PP) conical centrifuge tubes, each cell line divided equally into 4 tubes. Old media was removed from the cells to which 15 mL of fresh MA2 media at 45°C was added.

The banana cells were heat-shocked in a water bath at 45°C for 5 min after which the hot media was removed. With the exception of non-transformed controls, banana cells were resuspended in pre-induced Agro-suspensions (10 mL, OD_600_ = 0.623) enriched by 110 μL of Pluronic^®^ F-68 (0.02% w/v). For the non-transformed control, 10 mL of TMA1 media plus acetosyringone (200 μM) were added. Banana cells plus Agro-suspension were centrifuged two times at 900 rpm for 3 min and then shaken at room temperature for 2.5 hr. 800 μL of settled cell volume (scv) were spread on a nylon mesh and co-cultured on solid TMA1 media with 300 μM acetosyringone. Plates were sealed with Parafilm^®^ tape, wrapped in aluminium foil and incubated at 22°C for 3 days and thereafter washed three times with MA2 media supplemented with cefotaxime (300 µM). Cells of 800 μL scv were transferred to sterile nylon mesh and cultured in the dark on MA3 media for embryo formation. MA3 media was supplemented with hygromycin (pMDC32_Cas9_NktPDS) and kanamycin (pUBI: GUS) but no antibiotic selection for control (non-transformed cells). For transient assay, 200 μL of cells transformed with the pUBI: GUS construct were added to 800 μL of GUS stain and incubated overnight at 37°C.

Transformed banana cells were sub-cultured every two weeks on fresh hygromycin (15 μg/mL)-selective solid MA3 media in the dark for two months resulting in embryo formation. Embryos were transferred to hygromycin (15 μg/mL)-selective solid embryo regeneration media (ERM) in the dark for 1 month resulting in formation of shoots. The shoots were subsequently transferred to solid proliferation media. Wild-type controls underwent through the same process without hygromycin selection. The regenerated plantlets were visualised for the albino and variegated phenotypes due to the disrupted function of *PDS* gene. Leaf tissue was collected from selected shoots for downstream analysis.

### 5.5 Molecular characterisation of regenerated events

All primers used in the current study are described in **Supplementary Table 1**.

#### 5.5.1 Isolation of genomic DNA

Genomic DNA (gDNA) was extracted from 50 mg of freeze-dried tissue collected from all regenerated transgenic and non-transgenic plants using a modified cetyltrimethylammonium bromide (CTAB) method (Stewart and Via, 1993). Briefly, 50 mg of pulverised leaf sample was transferred into a 2 mL conical micro-tube and 1 mL of CTAB buffer was added. The mixture was briefly vortexed and incubated at 65°C for 30 min after which, 1 mL of chloroform:isoamyl alcohol (24:1) was added and mixed thoroughly by inversion. The reaction was centrifuged at 14,000 rpm for 5 min and the upper aqueous layer was transferred to a clean 2 mL micro-tube (Eppendorf). After addition of 2 μL of RNase A (1 μg/µL), the mixture was incubated for 1 hr at 37°C.

Two chloroform extractions were performed after which the resultant upper organic phase was transferred to a clean 1.5 mL micro-tube. An equal volume of isopropanol was added to each sample to precipitate gDNA and mixed thoroughly by inversion. The reaction was then allowed to stand at room temperature for 2 min and centrifuged at 14,000 rpm for 10 min. The supernatant was discarded and the pellet washed with 1 mL of ice-cold 80% (v/v) ethanol followed by centrifuging at 14,000 rpm for 5 min. Excess ethanol was removed, the pellet air-dried under vacuum for 10 min and subsequently resuspended overnight at 4°C in 50 μL of nuclease-free water. The gDNA purity, quality and concentration were confirmed by UV-Vis spectrophotometry.

#### 5.5.2 PCR analysis to confirm integration of *Cas*9 and *hpt*II genes

Integration of *Cas*9 and *hpt*II genes in the regenerated events was confirmed by primer pair OP1/OP2 and OP3/OP4 respectively. Each 10 μL PCR reaction contained 1X of OneTaq^®^ 2X Master Mix with standard buffer (New England Biolabs), 0.5 μM for each of the forward and reverse primers, 3 μL of nuclease-free water and 0.2 μg of DNA sample. Reactions were run in a C1000 Touch™ Thermal Cycler with Dual 48/48 Fast Reaction Module (BioRad) under the following conditions: initial denaturation at 95°C for 5 min; 32 cycles of 94°C for 30 sec, 55°C for 1 min, and 72°C for 1 min; followed by a final extension at 72°C for 7 min, then held at 8 °C. PCR products were then resolved through a 1.5% agarose gel stained with ethidium bromide.

#### 5.5.3 PCR analysis to detect band shifts

The mutations in the *PDS* gene were detected by band shift PCR analysis using the gene specific primers (OP5/OP6) flanking the two sgRNAs. The 50 μL PCR reaction contained 1X GoTaq^®^ Green Master Mix (2X), 1 μM of OP5/OP6, 0.2 μg of diluted DNA and 19 μL of nuclease-free water. PCR conditions were the same as described above and PCR products were resolved through a 1.5% agarose gel, stained with ethidium bromide to observe a shift in molecular size of bands.

#### 5.5.4 Sanger sequencing to detect mutations in selected gene-edited banana lines

To analyse the mutations at the target site in the gene-edited plants, Sanger sequencing was performed on selected regenerated events previously confirmed by band-shift PCR using OP5 primer. The PCR products were purified, diluted and sequenced using the Applied Biosystems^®^ 3130/3130xl Genetic Analyzer (Applied Biosystems, California) at the International Livestock Research Institute, Nairobi. The resultant sequences were aligned against the wild-type *PDS* gene using Geneious version 7.1.9 to identify and quantify insertion/deletion (indel) mutations.

### 5.6 Phenotypic and biochemical characterisation of gene-edited plants

#### 5.6.1 Phenotypic analysis

The regenerated plantlets were characterised for various phenotypes such as albino, variegated-albino and variegated which are due to the disrupted function of the *PDS* gene, as well as no albino-no variegated which is from wild type events.

#### 5.6.2 Carotenoid analysis and quantification

Total carotenoids were extracted from 25 mg of freeze-dried leaf samples of wild-type and gene-edited lines. Using stainless steel beads, the milled leaf tissue was crushed into a finer powder using a Mini Bead Beater (Biospec Products). Carotenoid extraction and quantification was carried out by HPLC-PDA at 450 nm as described by Buah et al. (2016). Briefly, acetone was added to homogenised leaf powder, vortexed and centrifuged for 5 min at 13,000 rpm at 4°C. The extraction was repeated twice, and supernatants were pooled and partitioned using petroleum ether/diethyl ether (2:1, v/v) and 1% (w/v) filter-sterilised NaCl. The mixture was centrifuged to isolate the organic phase, which was then vacuum-dried and stored at -20°C in preparation for HPLC-PDA analysis. Total carotenoids, pro-vitamin A carotenoids (pVACs) and β-carotene equivalents (BCE) were quantified and expressed as micrograms per gram of dry weight (µg/g DW).

### 5.7 Statistical analysis

All experiments were performed in duplicates. Data from the carotenoid quantification experiment were compared between gene-edited lines and wild-types. Gene-edited lines with zero carotenoid values were excluded from statistical analysis, as they do not contribute to variability. Results were presented as mean ± standard deviation (SD). Statistical analysis was performed using one-way Analysis of Variance (ANOVA), followed by Dunnett’s multiple comparison test to assess significant differences between gene-edited lines and wild type. All analyses were conducted using GraphPad Prism v8.0.1, with significance determined at P<0.05.

## Data Availability

All data generated during this study is embedded in this article. Additional data is available from the corresponding author upon request.
