# Correction: Efficient CRISPR/Cas9-mediated genome editing of *phytoene desaturase* in Musa-AAA: a critical step for genetic improvement of East African highland bananas

**DOI:** 10.3389/fpls.2026.1737693

**Published:** 2026-01-29

**Authors:** Frank Kalungi, Julius Mulindwa, Samwel Kariuki Muiruri, Valentine Otang Ntui, Abubakar Sadik Mustafa, Priver Bwesigye Namanya, Jerome Kubiriba, Leena Tripathi, Alex Barekye, Jimmy Moses Tindamanyire

**Affiliations:** 1 National Agricultural Research Laboratories, National Agricultural Research Organisation, Kampala, Uganda; 2 Department of Biochemistry and Systems Biology, College of Natural Sciences, Makerere University, Kampala, Uganda; 3 International Institute of Tropical Agriculture (IITA), Nairobi, Kenya; 4 Department of Plant Sciences, Microbiology and Biotechnology, College of Natural Sciences, Makerere University, Kampala, Uganda

**Keywords:** CRISPR/Cas9, Banana, PDS, Genome-editing, Carotenoids, Nakitembe, guide-RNA

Samwel Kariuki Muiruri, Valentine O. Ntui and Leena Tripathi were omitted as authors in the published article. The correct author list reads: “Frank Kalungi, Julius Mulindwa, Samwel Kariuki Muiruri, Valentine O. Ntui, Abubakar Sadik Mustafa, Priver Bwesigye Namanya, Jerome Kubiriba, Leena Tripathi, Alex Barekye and Jimmy Moses Tindamanyire”. The affiliation **“**International Institute of Tropical Agriculture (IITA), Nairobi, Kenya” has been included as affiliation 3.

The Author Contributions Statement has been corrected to read:

“FK: Conceptualization, Data curation, Formal Analysis, Investigation, Methodology, Software, Writing – original draft, Writing – review & editing. JM: Methodology, Supervision, Validation, Writing – review & editing. SM: Perform sequencing & Data Analysis. VN: Construct Preparation, Supervision. AM: Methodology, Supervision, Validation, Writing – review & editing. PN: Funding acquisition, Project administration, Resources, Supervision, Writing –review & editing. JK: Funding acquisition, Project administration, Writing – review & editing. LT: Methodology, Sequencing and Data Analysis, Supervision. AB: Project administration, Resources, Writing – review & editing. JT: Conceptualization, Funding acquisition, Investigation, Methodology, Project administration, Resources, Supervision, Validation, Visualization, Writing – review & editing.”

The original version of this article has been updated.

