# Supplementary material for: Efficient CRISPR/Cas9-mediated genome editing of phytoene desaturase in Musa-AAA: a critical step for genetic improvement of east African highland bananas
Source: Front Plant Sci. 2025 Sep 16;16:1677409. doi: 10.3389/fpls.2025.1677409 (PMC12479461; doi:10.3389/fpls.2025.1677409)
Supplement: Supplementary file 1 [file Table1.docx]

**Supplementary information**

**Table 1.** Primers and sgRNAs used in this study

| **Code** | **Primer name** | **Sequence (5'>3')** | **Application** |
| --- | --- | --- | --- |
| OP1 | *Cas9*-F | TCTCCTCGATCATCTCCCTATC | Amplification |
| OP2 | *Cas9*-R | CCTTACTACGTTGGTCCTCTTG |  |
| OP3 | Hyg-F | CAGTTTGCCAGTGATAC |  |
| OP4 | Hyg-R | GAATCTCGTGCTTTCAG |  |
| OP5 | BS-F | GCATTACCAGTAAAGCAAA | Amplification, sequencing |
| OP6 | BS-R | CCCAGAGGAAGATTTCCTT |  |
| sgRNAs*, BsmBI adaptors* (purple) and flanking nucleotides (green) | | |  |
| OP7 | gRNA1_PDS_F | GTGTGTATCAATGATCGCTTGCAA | Cloning, amplification |
| OP8 | gRNA1_PDS_R | AAACTTGCAAGCGATCATTGATAC | Cloning |
| OP9 | gRNA2_PDS_F | GTGTGTGGAGGGCAAGCTTATGTGG |  |
| OP10 | gRNA2_PDS_R | AAACCCACATAAGCTTGCCCTCCAC | Cloning, amplification |
| OP11 | P131-P133C_R | AATTGCCCTTCGAAGGGACA | Amplification |

**Table** **2:** Carotenoid content of selected regenerated lines

| **Line** | **Phenotype** | **Lutein**  **(μg/g DW)** | ***α*-carotene**  **(μg/g DW)** | ***trans β*-carotene**  **(μg/g DW)** |
| --- | --- | --- | --- | --- |
| H-W | Green | 133.98± 5.61 | 7.51±0.18 | 13.19±1.49 |
| H30 | Variegated | 49.87±0.41 | 3.78±0.15 | 6.13±1.39 |
| H31 | Albino and variegated | 0.49±0.05 | 0 | 0 |
| N-W | Green | 98.42±5.10 | 7.20±0.29 | 11.18±0.51 |
